# S-BIRD: A Novel Critical Multi-Class Imagery Dataset for Sewer Monitoring and Maintenance Systems

**DOI:** 10.3390/s23062966

**Published:** 2023-03-09

**Authors:** Ravindra R. Patil, Mohamad Y. Mustafa, Rajnish Kaur Calay, Saniya M. Ansari

**Affiliations:** 1 Faculty of Engineering Science and Technology, UiT The Arctic University of Norway, 8514 Narvik, Norway; 2 Department of E & TC Engineering, Ajeenkya D Y Patil School of Engineering, Pune 411047, India

**Keywords:** sewer monitoring, S-BIRD dataset, object detection, computer vision, YOLOX training, AI techniques

## Abstract

Computer vision in consideration of automated and robotic systems has come up as a steady and robust platform in sewer maintenance and cleaning tasks. The AI revolution has enhanced the ability of computer vision and is being used to detect problems with underground sewer pipes, such as blockages and damages. A large amount of appropriate, validated, and labeled imagery data is always a key requirement for learning AI-based detection models to generate the desired outcomes. In this paper, a new imagery dataset S-BIRD (Sewer-Blockages Imagery Recognition Dataset) is presented to draw attention to the predominant sewers’ blockages issue caused by grease, plastic and tree roots. The need for the S-BIRD dataset and various parameters such as its strength, performance, consistency and feasibility have been considered and analyzed for real-time detection tasks. The YOLOX object detection model has been trained to prove the consistency and viability of the S-BIRD dataset. It also specified how the presented dataset will be used in an embedded vision-based robotic system to detect and remove sewer blockages in real-time. The outcomes of an individual survey conducted at a typical mid-size city in a developing country, Pune, India, give ground for the necessity of the presented work.

## 1. Introduction

An underground sewerage system is an essential feature of town planning as it transports the wastewater away from its source for safe disposal in the environment with minimum impact on the surroundings. However, underground pipe systems have maintenance problems. Sewer blockages and various damages such as cracks, fractures, joint displacement, etc. all can cause overflow, leaching of sewage into soil and interference with drinking water supply lines. Poor maintenance also leads sewer pipes to deteriorate early.

Therefore, it is important for any responsible authority to ensure that sewers are in good condition and run properly. The Ministry of Housing and Urban Affairs conferred Standard Operating Procedure (SOP) for cleaning sewers and septic tanks in November 2018 [1]. Regular inspections are necessary to identify any event of crack or blockage so that corrective measures are taken in time to avoid a crisis. In the past, manual inspection was often used followed by circuit television (CCTV) which has been one of the most used methods in the US and European municipalities in recent decades. However, these methods are labor-intensive and error-prone.

Artificial Intelligence (AI) is used in computer vision technology that consists of intelligent algorithms to interpret meaningful digital information from images and videos, which, when combined with automated robotic systems, provide powerful vision and intelligence to detect various sewer problems and to plan corrective actions. However, training AI-based *Deep Neural Object Detection Models* and achieving sewer inspection objectives based on them requires large amounts of appropriate and labeled data. A dataset is a collection of featured and significant information in any field that is used to learn AI models for purposes such as detection, classification, regression, clustering, segmentation, etc. Data is usually in the form of images, text, numbers, time series, graphs, etc. The performance of the best detection model trained using a poor dataset is always inferior to the performance of a poor detection model trained using a highly featured and quality dataset. At the center of every object detector, whether single-stage or two-stage, is a classifier that secures the identities of all desired object classes. Clearly, the accuracy rate and performance of any detection model are highly dependent on the quality of the input imagery dataset.

Therefore, relevant dataset collection is a very important prerequisite for any AI model to predict outcomes with the desired accuracy and also has emerged as a prominent research theme in respective research communities. This involves data acquisition or collection, appropriately labeling the data and finally enhancement of obtainable data or models [2]. Due to the open-access research policy of many funding agencies, a large amount of data pertaining to many fields is available on various platforms. In many instances data may be available from data-sharing platforms like DataHub [3], Kaggle datasets [4], Mendeley Data [5], etc. and data searching platforms like Google Dataset Search [6], IEEE DataPort [7], etc. After tackling several challenges in data search, a researcher can succeed in obtaining the required dataset [8]. However, the European Commission recognized the difficulties in obtaining and tracing open data in 2011 and started to regulate data publishing activities in Europe [9]. Six snags in obtaining and tracing open data were identified: deficient details about the existence and accessibility of data, ambiguity about data ownership by public authorities, ambiguity about reuse terms, critical nature and cost of data, complex licensing processes and restrictive fees, specific reuse agreements with commercial members and reuse restrictions for state-owned companies.

**Figure 1:**
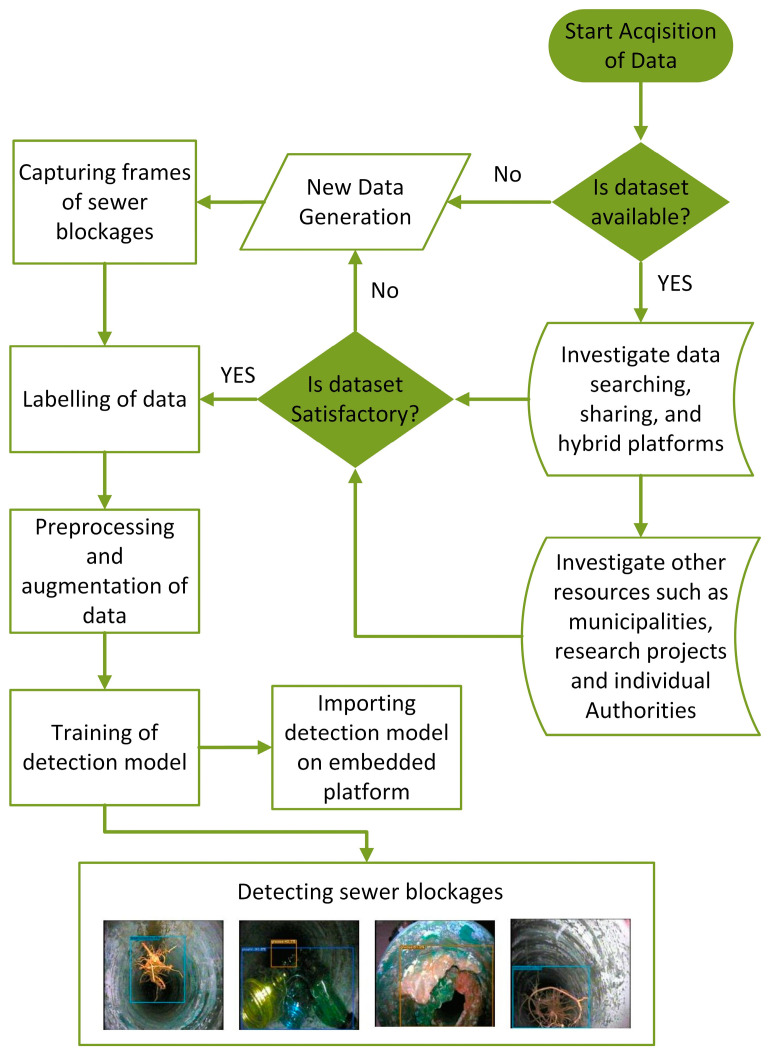
Workflow diagram of the presented S-BIRD dataset.

Specifically, data acquisition includes tasks such as searching, augmenting and generating as needed, and in our case, the dataset is not only created due to unavailability but also prepossessed, augmented and labeled individually for classification and detection tasks. Manual or automated techniques are used for dataset generation, while synthetic data is generated to fill the lacking portion of the dataset. A standardized or benchmark dataset is always a central aspect to obtain the best-fit learning models and the application of transfer learning techniques with the developed dataset plays an important role in the advancement of AI-based models [10]. In computer vision, a dataset of digital images containing object class information is grouped as needed into a training set, validation set, and test set to serve as input to a detection model for learning, evaluation, and testing purposes, respectively. A workflow with decision-making for the S-BIRD dataset presented in this paper is shown in Figure 1, which displays the process from generation requirements to the training results.

In this paper, a new critical multi-class imagery dataset S-BIRD (Sewer-Blockages Imagery Recognition Dataset) is presented to identify sewer blockages caused by grease, plastics and tree roots. The lack of a standardized matrix for algorithms applied in the real-world development of sewer monitoring and maintenance systems is a critical issue, and the submitted dataset addresses this. So, the S-BIRD sets the standard for detection outcomes in real-time scenarios. Validation results of the S-BIRD dataset are given and development on an embedded vision platform to overcome actual sewer blockages problem is considered. In the conferred work, all computer vision and model training operations are implemented using Python programming, OpenCV, PyTorch framework, and some other machine learning libraries on the DGX workstation system including the Linux platform. Both the presented dataset and the corresponding results highlight the importance and necessity of such research work for the treatment of wastewater sewer blockages.

## 2. Needs of the S-BIRD Dataset

In earlier work, a survey on sewer robotic systems and computer vision practices in sewer inspection works was carried out and that gave information about practical issues concerning sewerage systems under the Pune Municipal Corporation (PMC), India [11]. It was concluded that sewer blockage is the main issue of sewers in Pune and to date, there is no robust algorithm and robotic system available for both real-time detection and removal of sewer pipe blockages.

Unlike many Western countries, India has single sewer lines for both sewage and stormwater. Thus, this combined drainage system is a big problem, particularly for cleaning and removing blockages.

In order to develop the function of detecting and identifying sewer blockages in real time, authenticated datasets are a prerequisite. Thus, all available means were used to search for datasets. Several municipalities and various authorities were also contacted for relevant data information, but no concrete work and datasets that may be used for real-time detection of sewer blockages were available. Furthermore, it was not possible to acquire a specific dataset for Indian conditions focusing on the issue of sewer blockages. The harmful, unhygienic and foul smell of a sewer environment is always a major concern when capturing frames of sewer problems for dataset generation. It is appropriate to imply that independent binding, copyright or confidentiality issues relating to earlier works are also responsible for the unavailability of the datasets.

Sewer blockages are mainly caused by grease, plastic and tree roots. Other elements inside the sewer mix up with the black water and become difficult to identify. So, other elements are usually treated as a blackish sewer blockage, which is identified as black grease in the dataset. We also considered imagery data of grease, plastic and tree roots as mentioned above in the dataset S-BIRD, which is used for training of object detection model to locate and recognize the sewer blockages in real-time. 

**Figure 2:**
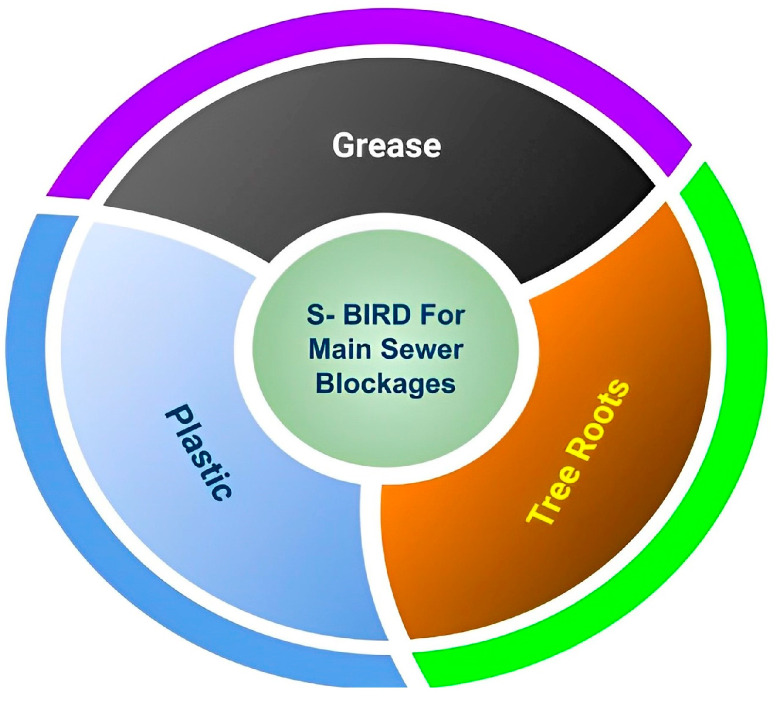
S-BIRD dataset for main sewer blockages.

Obviously, blind systems cannot be as efficient as vision-based sewer robotic systems. Figure 2 shows the concept of constructing the S-BIRD dataset that takes grease, plastic and tree roots into account.

## 3. Tools in S-BIRD Dataset Creation

In this section, the tools involved in creating the S-BIRD dataset are provided for detailed viewing.

### 3.1. Sewer Pipeline

**Figure 3:**
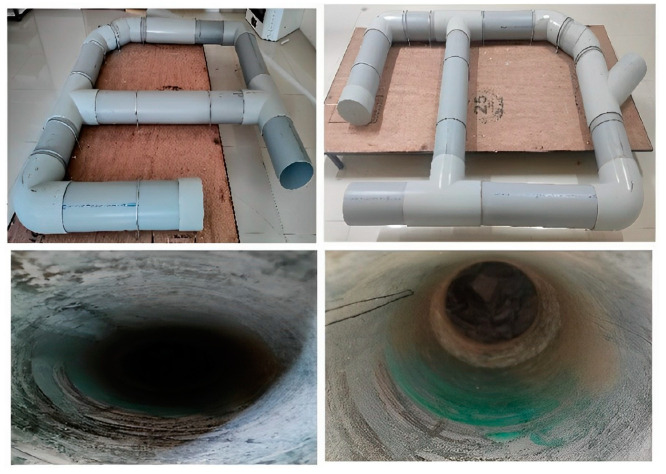
Constructed sewer pipeline.

In an unhygienic, muddy and smelly sewer pipe environment due to sewage, toiletry, sanitation, and stormwater from combined drainage systems, capturing real-time frames of sewer issues was a very difficult task for an individual. For simulating a sewer network, PVC pipelines of 200 mm diameter, which are widely used in residential sewers, were used to construct a typical sewer network. The constructed sewer pipeline is shown in Figure 3. 

In this case, there is no big difference between a real sewer environment and a laboratory setup or simulated sewer network. Exactly the same blockage types with inherent nature have been created inside the sewer network consisting of all featured information. The only difference was that the simulated sewer network did not have the stench and noxious atmosphere. The detection model trained using the developed S-BIRD dataset in the respective sewer network is capable to work in practical situations.

### 3.2. Sewer Inspection Camera

**Figure 4:**
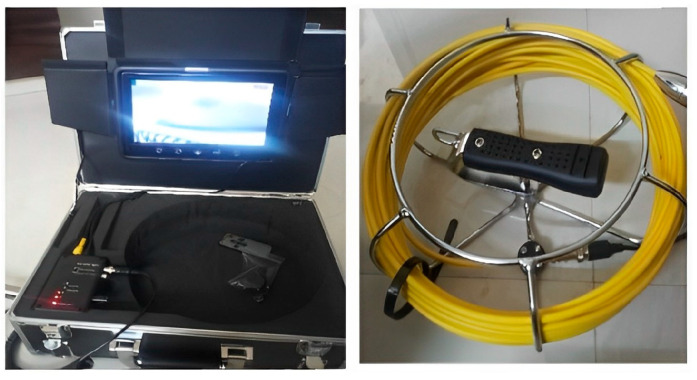
Watertight sewer camera.

Real-time frames of sewer barriers that include grease, plastics, and tree roots are captured by the watertight sewer camera shown in Figure 4, and its characteristics are given in Table 1.

This camera sensor is capable of capturing real-time frames at different angles not only for the intended aspect ratio but also for varying brightness due to attached modifiable white LEDs.

## 4. A Novel S-BIRD and Corresponding Results 

This section discusses compiled imagery data (Section 4.1), its arithmetic details (Section 4.2), preprocessing and augmentation techniques applied to captured frames (Section 4.3), and annotated heatmap and object count histograms (Section 4.4).

### 4.1. Imagery Data Collection

**Figure 5:**
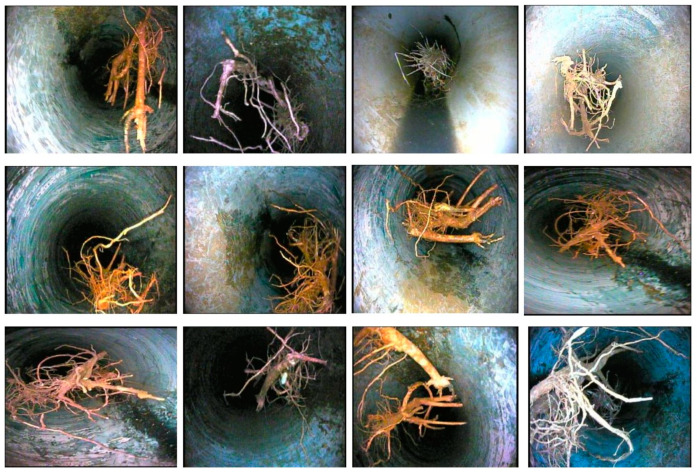
Tree root blockage frames in the S-BIRD dataset.

All images of sewer blockages are captured under different lighting conditions and from different angles to gather the necessary perceptions and features. Figure 5 reveals some blockage frames of tree roots in the newly created dataset.

**Figure 6:**
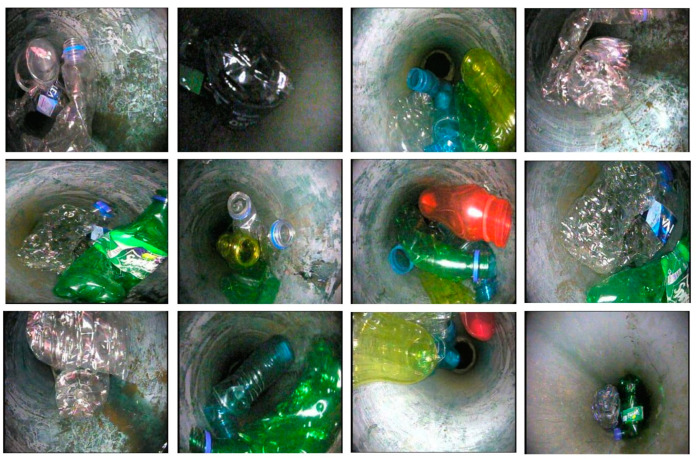
Plastic blockage frames in the S-BIRD dataset.

Dissimilar colored plastic is captured in the picture and key information for the detection and recognition task is achieved as shown in Figure 6.

There could be other elements within the black sewage mass such as plastic bags or other debris, but they look completely blackish as they are often mixed with black water and grease.

**Figure 7:**
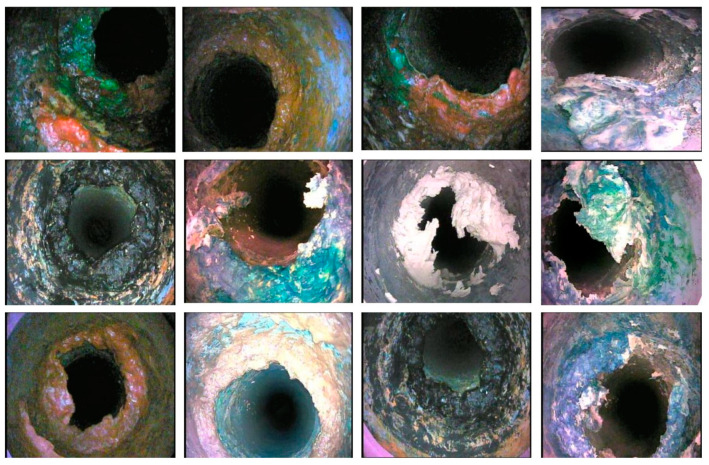
Grease blockage frames in the S-BIRD dataset.

Figure 7 exhibits grease blockage frames capturing diverse and significant colored information. There are a number of sources for grease-type sewer blockages which mainly include wastage from domestic and high- or low-density production plants that produce huge chemical and processed waste.

### 4.2. Arithmetic Details of Captured Frames

The arithmetic details of the captured frames are listed in Table 2 for further implementation. Certainly, annotating the objects in each captured frame was time-consuming but the task was still performed individually with high skill and accuracy without labeling errors. The annotations contain information about the location, i.e., center x, center y, width, height and class of objects present in each frame of the S-BIRD dataset. 

**Figure 8:**
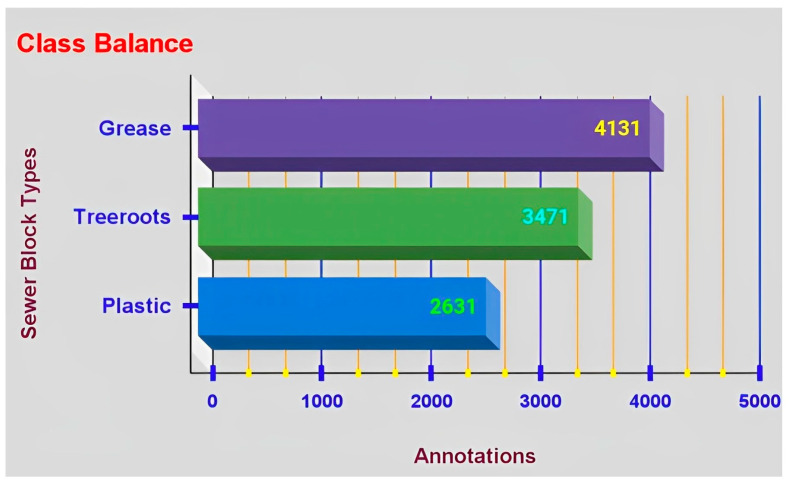
Annotation figures for class (sewer blockage type) balance.

Figure 8 stipulates the total number of annotations for class balance, i.e., annotations for each sewer block type and these are 4131 for grease, 3471 for tree roots and 2631 for plastic.

**Figure 9:**
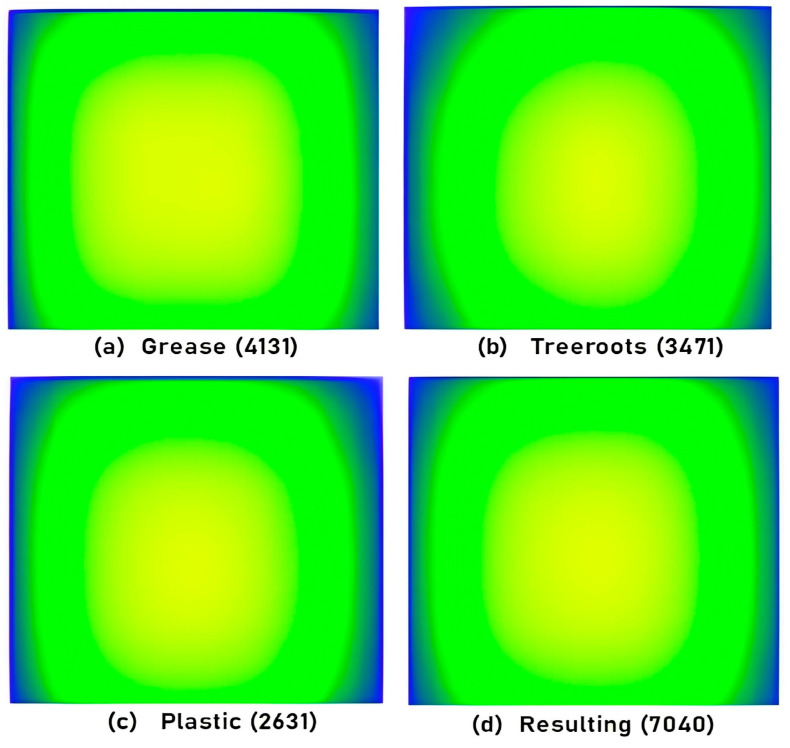
Annotation heatmap details for captured frames.

The location of annotations, i.e., bounding boxes for considered blockage types in all captured frames is shown by heatmap in Figure 9. A heatmap represents informative data in a graphical or two-dimensional form where a color-coding system is used to represent values, and in the above heatmap, values are annotation details. It confers a quick visible summary to perceive the intricate nature of the dataset. Here, the correlation between annotated values is made easier to understand using colors in a heatmap compared to numerical tables. The yellow color denotes a highly positioned region of annotations whereas the light green color indicates lower positioning. All depicted heatmaps show that the locations of annotations are mostly in the center of the frames of object classes. 

**Figure 10:**
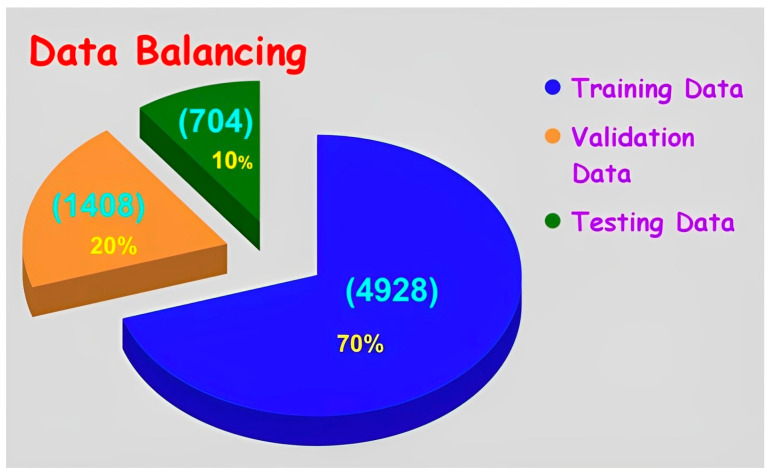
Imagery data balancing of particular sewer blockage type.

The imagery data is balanced into three groups such as training data with 4928 frames (70%), validation data with 1408 frames (20%) and testing data with 704 frames (10%) as shown in Figure 10.

Table 3 provides annotation details for the classes in the training data.

### 4.3. Preprocessing and Augmentation Techniques

Here, two preprocessing techniques have been implemented on captured frames such as auto-orientation of pixel data, i.e., discarding the EXIF rotation and validating the pixel sort as well as resizing to 416 × 416 (px) by stretching the frame without losing source frame information. An image preprocessing benefits to reduce model training time and speed up inference of detection models.

Here, two preprocessing techniques have been implemented on captured frames such as auto-orientation of pixel data, i.e., discarding the EXIF rotation and validating the pixel sort as well as resizing to 416 × 416 (px) by stretching the frame without losing source frame information. Image preprocessing benefits from reduced model training time and sped-up inference of detection models.

**Figure 11:**
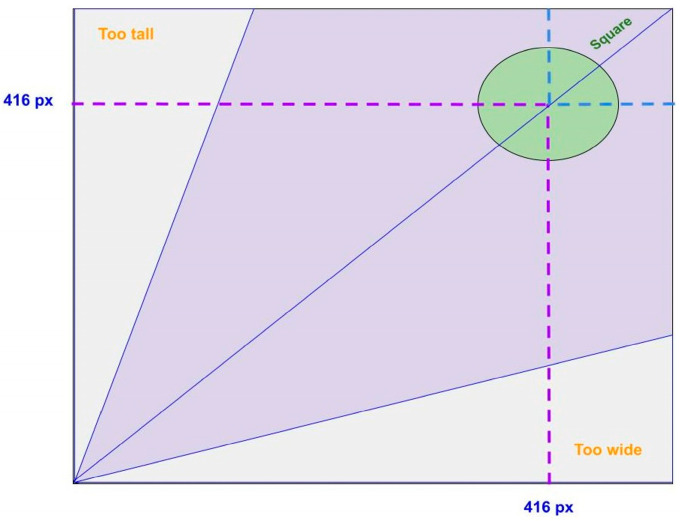
Aspect ratio distribution graph.

Figure 11 shows the aspect ratio distribution graph for the S-BIRD dataset and makes clear that all frames are 416 × 416 (px), i.e., square in size.

Further, image-level augmentation techniques have also been implemented to generate new training instances from existing training data.

**Figure 12:**
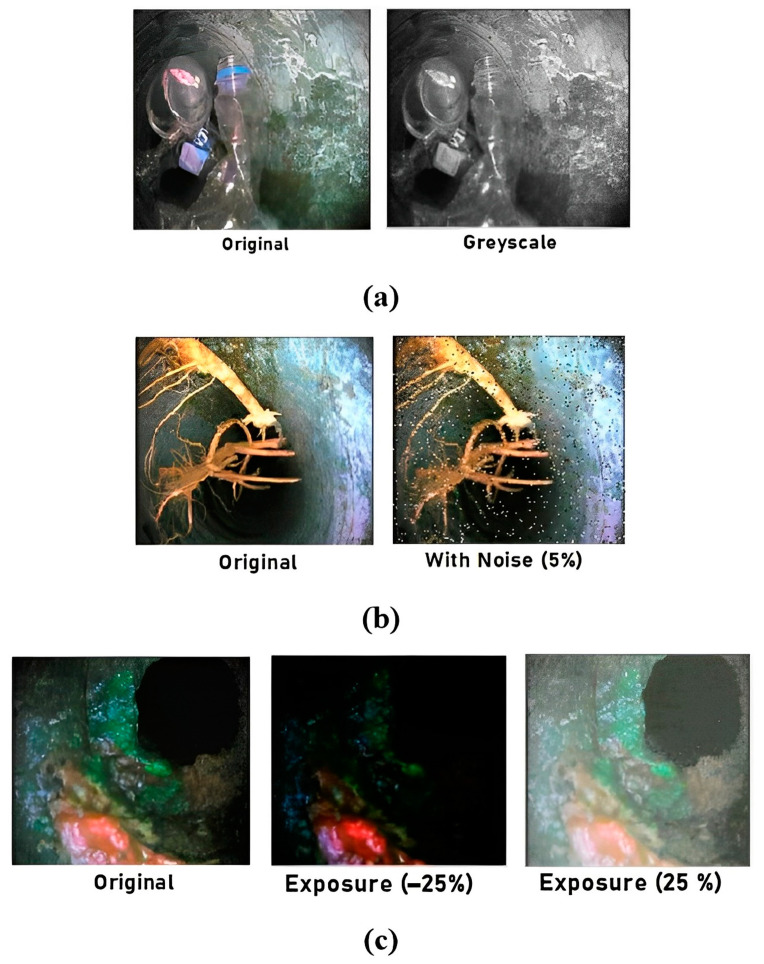
Visual upshots of standard augmentation techniques: (**a**) greyscaling, (**b**) salt and pepper noise, (**c**) random exposure.

Figure 12a shows the output frame of the gray scaling applied 25 percent to the input training frame which helps to increase the training variation but does not remove the color information when making inferences. Salt and pepper noise, also known as impulse noise, is applied to 5 percent of the pixels of the input frames as shown in Figure 12b which helps the detection model to turn out to be more flexible for camera artifacts through training. This noise involves adding some bright pixels to dark regions and some dark pixels to bright regions of the frames. It also helps to prevent adverse effects and avoid overfitting.

To strengthen the detection model against light and camera setting changes, random exposure adaptations were instigated between −25 and +25 percent for the input frame as shown in Figure 12c.

**Figure 13:**
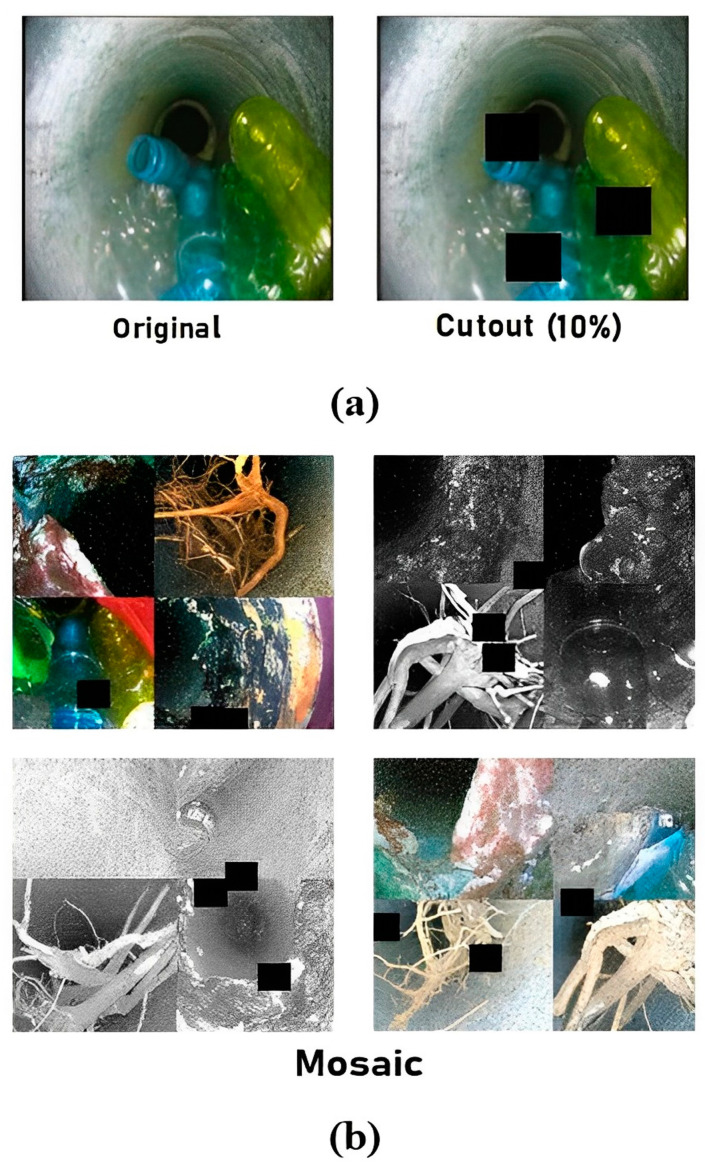
Visual upshots of advanced augmentation techniques: (**a**) cutout and (**b**) mosaic.

Two advanced augmentation techniques, namely cutout and mosaic, were exploited as shown in Figure 13a and Figure 13b, respectively. Adding cutouts to training frames is extremely useful for the detection model to be strong against the object occlusion state. For this, three cutouts were inserted in 10 percent of each of the total sizes of the input frames. Next, the mosaic technique helps the detection model to work well on small objects by joining several images from the training set in collage [12]. In this, four different sewer block frames were added in a single frame.

Augmentation techniques facilitate enhancing the efficiency of the object detection model by increasing the number and variegation of learning instances and related annotations. These techniques also reduce training time and costs for search detection models. So, discrete output versions have been generated for source frames.

In Table 4, the numerical details of training frames in S-BIRD are demonstrated after applying preprocessing and augmentation techniques.

**Figure 14:**
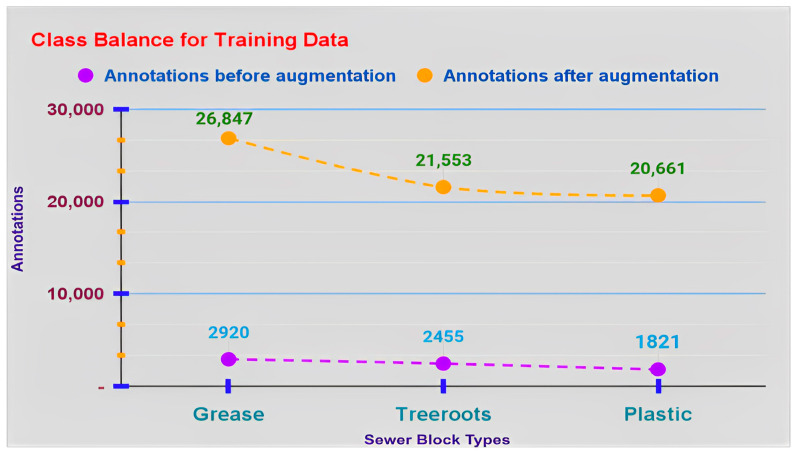
Annotation details for every single class in training data after image level augmentation.

The graph in Figure 14 shows the escalated annotations for each sewer block type in S-BIRD’s training data, after using annotation techniques. Now there are 26,847 annotations for grease, 21,553 for tree roots and 20,661 for plastics making a total of 69,061 augmented annotations, i.e., bounding boxes. Total annotations have increased by 61,865, i.e., 859.714%. Both preprocessing and augmentation techniques have been implemented using OpenCV, a computer vision and machine learning library, along with Python programming on the Linux platform from scratch to achieve the desired results.

### 4.4. Annotated Heatmap and Object Count Histogram

**Figure 15:**
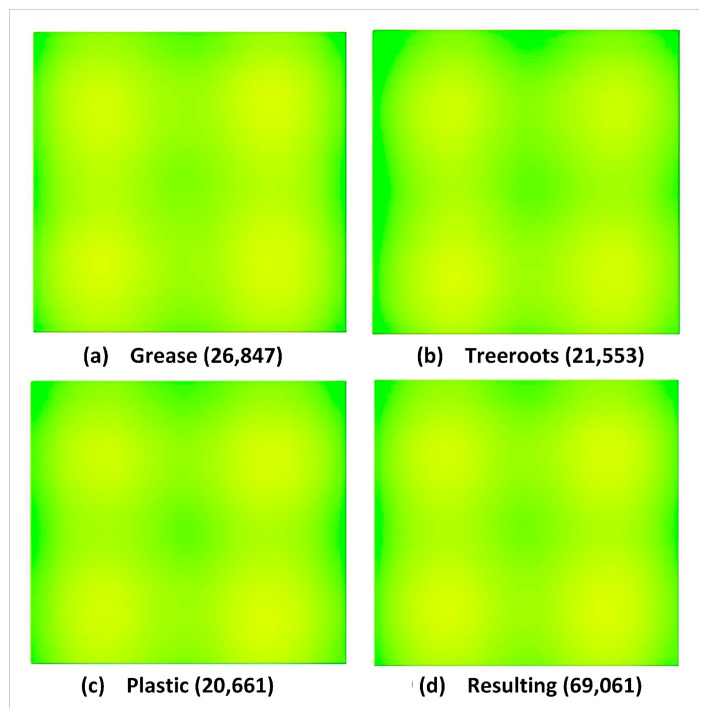
Annotation heatmap details for all classes.

Two important parameters, namely the annotated heatmap and the object count histogram have been examined to assess the efficacy of the training data. The location of the entire annotations for grease, plastic and tree roots in S-BIRD’s training data is illustrated by heatmaps in Figure 15. The specified heatmap informs us of the utmost generic position and weightage of all the annotations for revealed classes. From the color information of the heatmaps, it can be seen that most of the annotation locations are at the far left and right of both the top and bottom sides of the frames of object classes.

**Figure 16:**
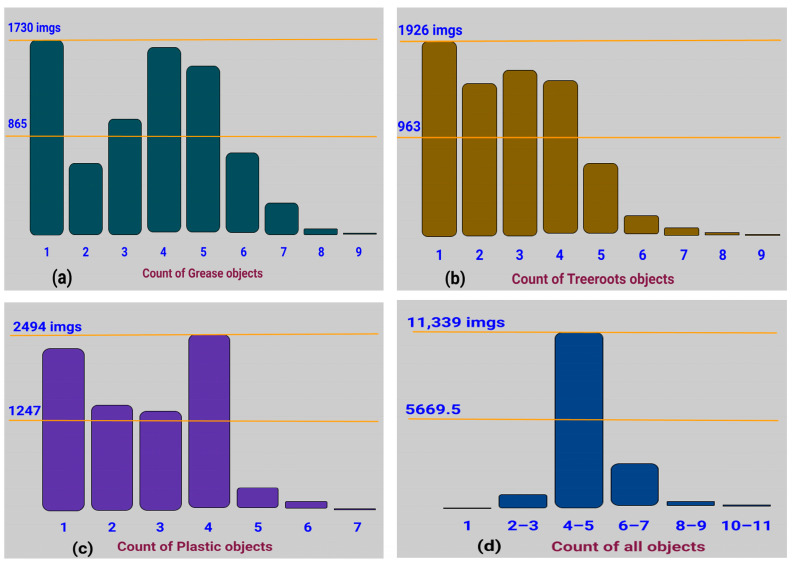
Object count histogram for: (**a**) grease, (**b**) tree roots, (**c**) plastic, and (**d**) all classes.

A histogram is a chart that plots numeric data into bins represented by individual columns. Figure 16 details the number of frames on the y-axis and bins, i.e., the number of corresponding objects for all classes on x-axis, with the help of the object count histogram.

The number of objects, i.e., annotations for both grease and tree roots blocks are up to nine shreds as shown in Figure 16a,b. There is obviously one grease object for 1730 frames andfour to five grease objects for 1400 to 1600 frames as given in Figure 16a. In total, 1926 frames contain a single tree root object and about 1500 frames contained three to four tree root objects as shown in Figure 16b. The number of plastic objects varies up to seven shreds as shown in Figure 16c in which four plastic objects are in 2494 frames and perceptibly one plastic object in about 2200 frames.

Figure 16d represents the object count histogram of all classes where 11,339 frames contain four to five objects. It also shows details for a much lower aggregate overall for a single object in frames as compared to the ratio for 69,061 annotations. The findings obtained for both parameters such as the annotated heatmap and the object count histogram prove the high veracity and standard for each imagery data class in S-BIRD.

## 5. Training of Object Detection Model

### 5.1. Insight on Conformation of Object Detector Models

**Figure 17:**
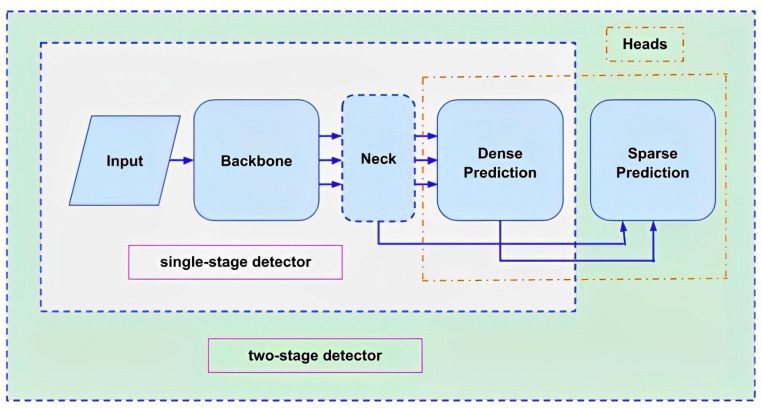
Conformation of object detector models.

Ordinarily, object detectors have two important segments, the backbone with pretraining to extract the features of input frames and the head which utilizes feature maps to predict classes and bounding boxes. Some layers are placed between the backbone and the head of recent object detectors to collect feature maps from distinct phases known as the neck. Object detectors with a backbone and densely predicted head are known as single-stage detectors, such as YOLO and SSD, while two-stage detectors have a backbone and head with dense and sparse predictions such as R-FCN, Faster R-CNN as shown in Figure 17. However, since single-stage detectors are faster than two stage detectors, they are used for multifarious real-time embedded applications. These object detectors embedded in robotic artifices are utilized to detect various faults in the sewerage system [13,14].

Table 5 lists some instances of the conformation parts in the object detector models.

The popular one-stage YOLO detection model is constantly being improved for better performance. An advanced version of the YOLO detection model is the recently introduced YOLOX which comprises three different basic embarkations, such as (a) anchor-free design which uses a center-based approach with each pixel detection mechanism for the selection of just one positive instance which then estimate four distances such as left, top, right, and bottom from positives to the border, i.e., prediction consists of a single 4D vector to encode the location of the bounding box at every foreground pixel, (b) decoupled head for classification and regression, and (c) advanced label allocation tactics namely SimOTA which lessen the training time and evade other clarifier hyperparameters in the SinkhornKnopp algorithm, making it faster and more efficient than its equivalents [19]. The performance of YOLOX has been improved with addition of mosaic and mixup augmentation. YOLOv3 and Spatial Pyramid Pooling (SPP) layers with Darknet53 are employed as baseline by YOLOX. This detection model of different sizes has attained consistent improvements against all compatible counterparts when tested on modified CSPNet backbone in addition to the Darknet53 backbone.

### 5.2. Training of YOLOX Using S-BIRD

So, the small YOLOX detection model in PyTorch framework allowing mobile deployment has been trained to detect the main types of sewer blockages such as grease, plastic and tree roots using the newly developed S-BIRD. Annotations for sewer block types in S-BIRD were implemented in Pascal VOC format as per the requirement to advance the training process. The Tesla V100-DGXS-32GB GPU workstation was used as a training platform via Docker Container with a defined image.

Table 6 makes available particulars on crucial traits in the YOLOX-s training process.

The results obtained for the timing and precision of the YOLOX-s trained model for S-BIRD are given in Table 7 and Table 8, respectively.

**Figure 18:**
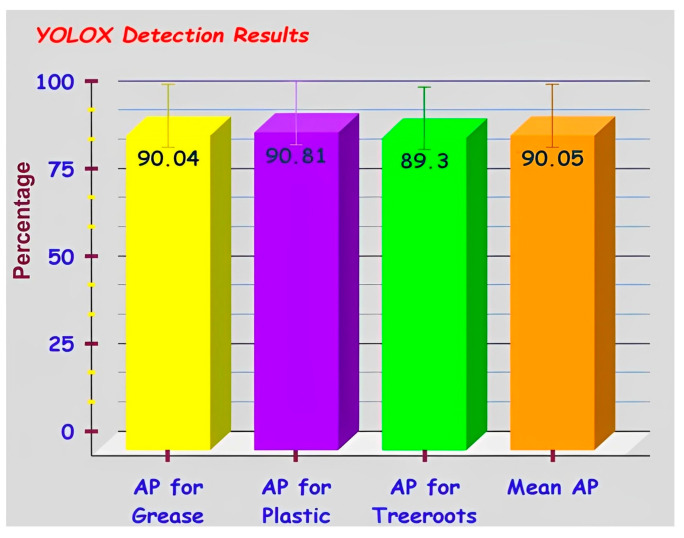
YOLOX detection results for all classes in S-BIRD.

**Figure 19:**
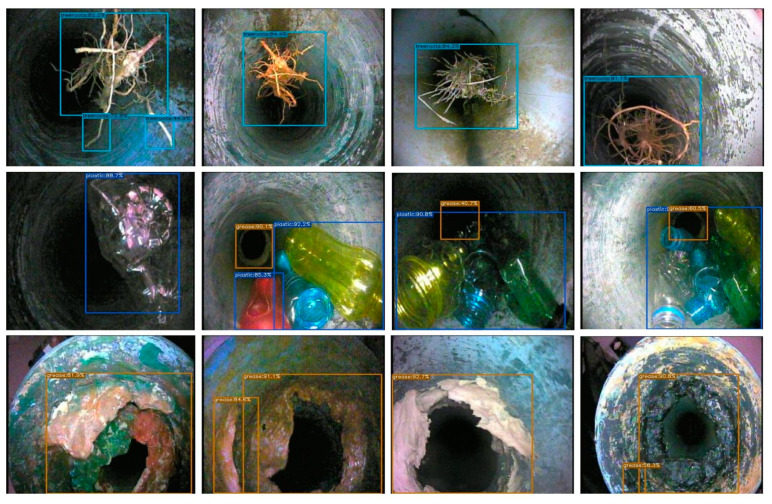
Visual upshots of detected tree roots, plastic and grease types of sewer blocks.

From Table 7 and Figure 18, YOLOX-s has achieved 90.04% AP for grease blocks, 90.81% AP for plastic blocks, 89.30% AP for tree root blocks, and 90.05% mean-AP computed at IoU (Intersection over Union) threshold 0.5. Another m-AP calculated over different IoU thresholds, from 0.5 to 0.95 with a step of 0.05 is 78.85%. The best-fit model is selected using cross-validation or rotation estimation technique [20]. The visual upshots of precisely detected sewer blocks such as tree roots, plastic and grease, are delineated in Figure 19. Of course, multiple sewer blockages in the same frame have also been considered for real-time detection purposes. Overall, the obtained results of the YOLOX-trained model prove the consistency and viability of the new S-BIRD dataset presented.

### 5.3. Embedded Vision with S-BIRD

The embedded vision is a pioneering and comprehensive platform for real-world visual implementations in the areas of home life equipment, health, daily services, security through detection and tracking, etc. [21,22]. So, the object detection model trained using S-BIRD will be a significant addition to existing or newly developed embedded vision-based sewer robotic systems.

**Figure 20:**
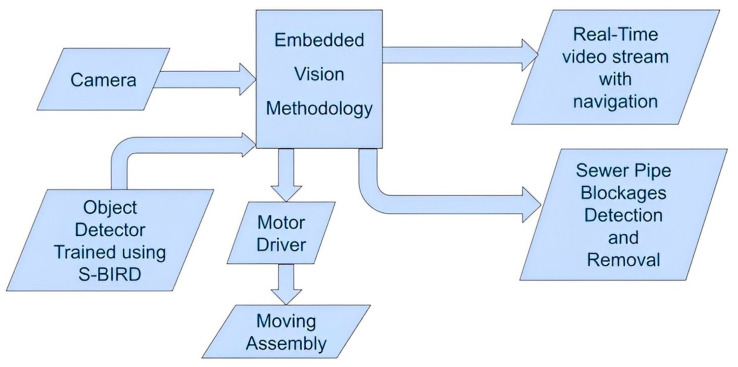
Block diagram of an automated system.

PIRAT [23], KARO [24], KURT [25], MAKRO [26], KANTARO [27], SIAR [28], etc. are some of the popular developments in the field of sewer robotics that serve the purpose of sewer inspection. Figure 20 shows the block diagram of an automated system that has a power-driven cutting tool to remove sewer blocks located by a detector trained using S-BIRD.

**Figure 21:**
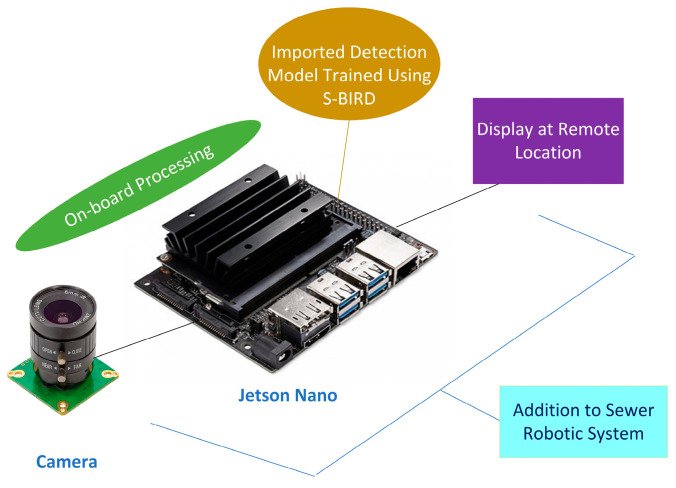
Embedded vision platform for sewer robotic system.

Here, Jetson nano has been selected as the embedded platform having a 4 GB GPU card of 128-Cuda cores and is suitable for running deep neural-network-based object detector models and for processing contiguous frames in real-time. Cameras such as a webcam, arducam, or raspicam are used to capture the surrounding frames for the purpose of navigation and processing, and then the output frames of detected sewer blockages are displayed on the screen to a remote location as shown in Figure 21.

In order to solve the recurring problem of underground sewer barriers in the practical world, a smart and comprehensive vision-based automation system with an AI detector trained using S-BIRD is certainly capable of meeting the needs of responsible authorities of any country.

## 6. Conclusions

In conclusion, a new critical multi-class imagery dataset S-BIRD which includes frames of main sewer blocks such as grease, plastic and tree roots is introduced to fulfill the need for implementing computer vision to automated robotic systems for identifying blockages in the sewerage pipes.

Arithmetic details for both compiled, as well as preprocessed and augmented data are discussed. The obtained results for preprocessing and augmentation demonstrate the increased number and variegation of learning instances and related annotations for the efficient performance of the object detection model. The procured details of heatmaps and object count histograms prove the high strength, veracity and standard for each imagery data class in S-BIRD.

The trained small YOLOX model achieved 90.04% AP for grease blocks, 90.81% AP for plastic blocks, 89.30% AP for tree root blocks, 90.05% Mean-AP at 0.5 IoU threshold, and 78.85% Mean-AP at 0.5 to 0.95 IoU thresholds for 300 epochs using S-BIRD. The relevant outcomes prove the consistency and viability of the new S-BIRD dataset presented. The object detectors trained using the presented S-BIRD will be a valuable addition to the existing or newly developed embedded vision-based sewer monitoring and maintenance systems for detecting sewer blockages in real-time scenarios.

## Figures and Tables

**Table 1:** Specifications of a utilized sewer camera.

Facets	Details
camera dimension	23 mm × 120 mm
camera light	12 modifiable white LEDs
watertight grade	IP68
vision angle	140 degree

**Table 2:** Arithmetical details of captured frames.

**Captured frames**	**Object Class (Sewer Blockage Type)**	**Captured Frames**
	Tree roots	2295
	Plastic	2392
	Grease	2353
Total frames	7040	
Annotations	10,233 (Average = 1.5 per frame)	
Average frame size	0.08 Megapixels	
Mean frame ratio	352 × 240 (wide)	
Angle of diagonal	0.598 radian = 34.3°	
Length of diagonal	426 pixels	
Aspect ratio Class	1.467:1	
Pixel density	9 pixels/mm or 230 pixels/inch	

**Table 3:** Annotations for training data.

Object Class (Sewer Blockage Type)	Annotations
Grease	2920
Tree roots	2455
Plastic	1821
Total	7196 (Average = 1.5 per frame)

**Table 4:** Arithmetical details of training frames in S-BIRD after preprocessing and augmentation.

Terms	Details
Total frames	14,765
Annotations	69,061 (Average = 4.7 per frame)
Average frame size	0.173 Megapixels
Mean frame ratio	416 × 416 (square)
Aspect ratio Class	1:1
Angle of diagonal	0.785 radian = 45°
Length of diagonal	588 pixels
Pixel density	12 pixels/mm or 290 pixels/inch

**Table 5:** Instances of conformation parts in the object detector models.

Conformation Parts		Details
Input		frames, multi-scaled frames, frame patches
Backbones		CSPDarknet-53 [15], Darknet53 [16], ResNet-50, ResNet-152, ResNet-10, GoogLeNet, Inception-ResNet-V2, EfficientNet-B0/B7, DetNet-59, ThunderNet, CBNet, VGG16, ViT, etc.
Neck		Bi-FPN, FPN, SFAM, PAN, etc.
Heads	Dense	YOLO [17], SqueezeDet, DetectNet, SSD, RetinaNet, MatrixNet, CenterNet, etc.
	Sparse	Mask R-CNN, R-FCN, Faster R-CNN [18], Cascade R-CNN, etc.

**Table 6:** Crucial traits in training.

Traits	Values
learning model	YOLOX-s
Annotation data type	VOC
max_epoch	300
batch_size	16
fp16	True
num_classes	3
Params	8.94 M
Gflops	26.64
depth	0.33
width	0.5
input_size	(640, 640)
random_size	(14, 26)
nmsthre	0.65
degrees	10.0
translate	0.1
scale	(0.1, 2)
mscale	(0.8, 1.6)
shear	2.0
warmup_epochs	5
weight_decay	0.0005
momentum	0.9

**Table 7:** Time results of the trained model.

Timing Parameters	Outturns (Milliseconds)
Average forward time	3.19 ms
Average NMS time	0.88 ms
Average inference time	4.07 ms

**Table 8:** Precision results of the trained model.

Class (Sewer Block Type)	Average Precision	Map_5095	Map_50
grease	0.9004	0.7885	0.9005
tree roots	0.8930		
plastic	0.9081		

## Data Availability

The research data will be made available on the request.
